# Content-Related Quality Control of Water- and Fat-Soluble Vitamins in Fortified Non-Alcoholic Beverages

**DOI:** 10.3390/nu16223872

**Published:** 2024-11-13

**Authors:** Žane Temova Rakuša, Robert Roškar

**Affiliations:** Faculty of Pharmacy, University of Ljubljana, Aškerčeva cesta 7, 1000 Ljubljana, Slovenia; zane.temova.rakusa@ffa.uni-lj.si

**Keywords:** energy drink, fortified food, HPLC, instant drink, juice, label claim, LC-MS, nutrition declaration, vitamin content, vitamin fortified water

## Abstract

Background/Objectives: Vitamin-fortified non-alcoholic beverages (VFNABs) are rising in popularity and availability. However, owing to their regulation as foods, there are also growing quality and safety concerns. Therefore, we aimed to provide an overview of the content-related quality of VFNABs on the Slovenian market. Methods: Vitamin contents in 50 VFNABs (29 waters, 5 juices, 12 energy drinks, and 4 instant drinks) were determined using validated methods based on liquid chromatography. Results: The results revealed several quality issues, which were determined in all four VFNAB types. These included an indication of at least one vitamin, present in a lower-than-significant amount, on the nutrition declaration in 64% of the tested VFNABs and vitamin contents outside the tolerance interval (65–150% of the label claim) in almost half of the cases (48.4%, *n* = 244). Since the disclosed quality issues are not only a reason for the misleading of consumers but may also pose safety risks for both individuals and public health, we further performed an overall assessment of the VFNABs as vitamin sources. The results revealed that the tested VFNABs not only fully cover but in several cases considerably exceed daily vitamin needs (up to 616% of the reference values), an effect which is further accentuated in children. Conclusions: The performed content-related quality control study undoubtedly highlight the need for stricter quality control and regulation and can be utilized as a foundation and recommendation for the manufacturers in terms of committing to and pursuing the production of VFNABs.

## 1. Introduction

Vitamins are a diverse group of organic compounds. They comprise water-soluble vitamins, i.e., vitamins C and B: thiamin, riboflavin, niacin, pantothenic acid, vitamin B6, biotin, folate, and cobalamin. They also include fat-soluble vitamins A, D, E and K. Each of them has a specific set of functions in the body. Since, the human body cannot synthesize them, except for vitamin D and niacin under certain conditions, vitamins are classified as essential micronutrients, whose sufficient and regular intake is needed to prevent deficiencies and maintain health at all stages of a person’s life [1,2,3]. Vitamin requirements change throughout the life cycle and are increased during pregnancy, lactation, growth, and in the elderly [3,4,5]. Increased vitamin requirements may also be a consequence of certain diseases, mostly associated with their impaired absorption (e.g., inflammatory bowel disease) [6], increased excretion (e.g., chronic kidney disease) [7], or the use of certain medicines (e.g., diuretics, oral contraceptives, statins) [8]. Sufficient vitamin intake is also crucial for maintaining and improving the body’s immune function, a fact which was especially exploited during the COVID-19 pandemic. After its outbreak, the consumption and sales of products containing individual vitamins (typically vitamins C, D, A, and E) and multivitamin products increased greatly in most countries (USA, UK, France, Poland, Slovenia), which was also reflected in their greater availability and diversity on the market in recent times [9,10,11].

Along with the increase in their availability and consumption, there are also growing quality, and consequently, efficacy, and safety concerns, which are supported by recent studies revealing the inadequate content-related quality of fortified foods (FF) [12,13,14] and enabled by regulatory oversights by the official authorities [11]. FFs sold in the EU are regulated as foods and as such must comply with the relevant food law EU and national regulations. In order to harmonize regulation on foods throughout the EU, Regulation (EC) No. 178/2002 of the European Parliament and the Council, passed on 28 January 2002, which laid down the general principles and requirements of food law, established the EFSA, and laid down procedures in matters of food safety, came into force. This regulation and its amendments impose safety criteria and require adequate and appropriate labeling, which applies to all foodstuffs, also including FFs, while proof of their quality is not necessary [15]. Since European legislation does not consider FFs to comprise a specific food category, there is no specific regulatory framework for FFs in EU food law. In the absence of proper regulations and control of FF, their quality may be questionable and depends on the efforts and activities of the manufacturers, especially towards vitamin stabilization, as specific vitamins (vitamin C, D, E, biotin, folic acid, etc.) are unstable and prone to degradation during FF manufacture and storage [16,17,18,19,20]. Instead of exploring and providing proper solutions, manufacturers often tackle the vitamin instability challenge by the use of overfortification [16,17,21,22,23].

Within this study, we focused on the quality control of non-alcoholic beverages, fortified with vitamins, which have recently become extremely popular, especially among adolescents, as a convenient source of vitamins [24,25,26,27]. Despite being advertised as a contribution to health maintenance and promotion, vitamin-fortified non-alcoholic beverages (VFNABs) can also cause adverse effects, particularly during growth and development, which may be associated with their inappropriate quality (significantly higher contents than declared on the label) and the consequent excessive intake of vitamins [25,26]. On the other hand, since vitamins are susceptible to degradation, their improper formulation, stabilization, manufacture, and storage could lead to their absence or presence in significantly lower amounts than declared. This may be associated with their intake being insufficient to meet human needs or overcome nutritional deficiencies [16,28]. Quality issues are thus not only a reason for the misleading of consumers, but may also pose certain safety risks. Being aware of this, and in the absence of relevant data on this subject, we evaluated the content-related quality of commonly consumed VFNABs (waters, juices, energy drinks, and instant drinks) on the Slovenian market by determining the contents of water- and fat-soluble vitamins. To the best of our knowledge, this is the first study to provide insight into the quality and safety of a broad sample of VFNABs with regard to vitamin content, which provides important information for the lay and professional public.

## 2. Materials and Methods

### 2.1. Chemicals and Reagents

The following fat- and water-soluble vitamin standards were purchased from Sigma-Aldrich (Steinheim, Germany): L-ascorbic acid (99.4%), thiamin hydrochloride (99.9%), nicotinamide (99.9%), pyridoxine hydrochloride (100.0%), calcium-D-pantothenate (99.0%), riboflavin (100.0%), riboflavin sodium 5′-phosphate (76.5%), cyanocobalamin (99.8%), and cholecalciferol (98.0%). D-biotin (100.6%), folic acid (97.8%), DL-alpha-tocopherol (97.0%), and DL-alpha-tocopherol acetate (99.3%) were purchased from Carbosynth (Berkshire, UK). HPLC-grade acetonitrile (ACN), methanol (MeOH), and tetrahydrofuran (THF) were purchased from Sigma-Aldrich (Steinheim, Germany). Sodium dihydrogen phosphate monohydrate, hydrochloric acid, ethylenediaminetetraacetic acid disodium dehydrate (EDTA), and formic acid were obtained from Merck (Darmstadt, Germany). Ultra-pure water (Milli-Q) was obtained through a Milli-Q^®^ Advantage A10 Water Purification System (Millipore Corporation, Bedford, MA, USA).

### 2.2. Instrumentation and Analysis

#### 2.2.1. HPLC (High-Performance Liquid Chromatography) Analysis

Water-soluble vitamins were analyzed on an ultra-high-performance liquid chromatograph Agilent Infinity 1290 system (Agilent Technologies, Santa Clara, CA, USA), equipped with a DAD detector and an EZChrom acquisition system, according to a validated and previously published stability-indicating method designed for their simultaneous analysis. We utilized a reversed-phase XSelect CSH C18 150 × 4.6 mm, 3.5 µm particle size column (Waters Corporation, Milford, MA, USA) operating at 40 °C, a mobile phase consisting of 25 mM NaH_2_PO_4_ × H_2_O with a pH = 4.0, and methanol in a gradient elution mode at a flow rate of 1 mL/min for their analysis. The detection wavelengths were as follows: 210 nm for calcium-D-pantothenate and D-biotin, 245 nm for thiamin, 260 nm for nicotinamide, 287 nm for folic acid, 290 nm for L-ascorbic acid and pyridoxine, 362 nm for cyanocobalamin, and 445 nm for riboflavin and riboflavin sodium 5′-phosphate [16].

Fat-soluble vitamins were analyzed on an Agilent 1100/1200 series HPLC system (Agilent Technologies, Santa Clara, CA, USA), equipped with a UV-VIS detector and ChemStation data acquisition system, according to a published, validated method for their simultaneous analysis. In brief, a reversed-phase Luna C18 150 × 4.6 mm, 5 μm particle size column (Phenomenex, Torrance, CA, USA) operating at 25 °C, and a mobile phase consisting of acetonitrile/tetrahydrofuran/Milli-Q (50:45:5, *v*/*v*/*v*) at a flow rate of 1 mL/min were used for their analysis. Their detection was carried out at 270 nm [22].

#### 2.2.2. LC-MS/MS (Liquid Chromatography–Tandem Mass Spectrometry) Analysis and Method Validation

Products containing biotin, folic acid, cyanocobalamin, cholecalciferol, and vitamin E (as alpha-tocopherol or alpha-tocopherol acetate) at low concentrations (near or below the quantitation limits of the above-described HPLC methods) were additionally analyzed on an Agilent Infinity 1290 system coupled to a 6460 triple quadrupole mass spectrometer with a Jet stream electrospray ionization source (Agilent Technologies, Santa Clara, CA, USA). Their chromatographic separation was performed on a Kinetex C18 50 × 2.1 mm and a 2.6 μm particle size column (Phenomenex, Torrance, CA, USA). The column temperature was maintained at 40 °C and the injection volume was 1 μL for biotin, folic acid, cyanocobalamin, alpha-tocopherol, and alpha-tocopherol acetate and 5 μL for cholecalciferol. The autosampler was kept at a constant temperature of 8 °C. The mobile phase contained a mixture of aqueous (0.1% formic acid, mobile phase A) and organic (methanol, mobile phase B) phases. Due to the significant difference in the retention of the analyzed water-soluble (biotin, folic acid, and cyanocobalamin) and fat-soluble vitamins (cholecalciferol, alpha-tocopherol, and alpha-tocopherol acetate), two chromatographic and MS/MS methods were used, keeping most of the chromatographic and MS parameters the same. Gradient elution (5–25% B, flow rate 0.5 mL/min, run time 5 min) was used for the analysis of biotin, folic acid, and cyanocobalamin, and isocratic elution (92% B, flow rate 0.5 mL/min, run time 5.5 min) was used for the analysis of cholecalciferol, alpha-tocopherol, and alpha-tocopherol acetate. The common MS/MS instrument conditions used for the analysis of biotin, folic acid, and cyanocobalamin are provided below, along with the differencing conditions for the analysis of cholecalciferol, alpha-tocopherol, and alpha-tocopherol acetate, which are provided in parentheses: a gas temperature of 275 °C and a flow of 5 L/min (10 L/min), a sheath gas temperature of 320 °C and a flow 11 of L/min, a nebulizer pressure of 45 psi (30 psi), a capillary voltage of 2.1 kV (5.0 kV), and a nozzle voltage of 0 V (2.0 kV). The two-positioned diverter valve was set to an MS between 2.9 and 4.2 min (1.2 and 4.5 min). The remaining MS/MS parameters are provided in Supplementary Materials (S1. LC-MS/MS analysis).

Both LC-MS/MS methods were validated according to ICH guidelines Q2(R2) in terms of linearity, detection limit (DL), quantitation limit (QL), precision, accuracy, and sample stability [29]. The preparation of samples for the validation of the LC-MS/MS method and LC-MS/MS method validation criteria are described in Supplementary Materials (S2., entitled ‘Preparation of samples for validation of the LC-MS/MS methods’, and S3., entitled ‘LC-MS/MS method validation’).

### 2.3. Commercial Products

VFNABs containing one or more vitamins were purchased locally on the Slovenian market between 2022 and 2024. The tested products comprised 29 waters, including Vitamin Well (Vitamin Well AB, Stockholm, Sweden), First (Fructal d.o.o., Ajdovščina, Slovenia), Dana (Dana, d.o.o., Mirna, Slovenia), Jana Vitamin (Jamnica plus, d.o.o., Zagreb, Croatia), Zala (Pivovarna Laško Union d.o.o., Ljubljana, Slovenia), OK (Mercator-Emba Proizvodnja Hrane, d.o.o., Logatec, Slovenia), REfresh (Petrol d.d., Ljubljana, Slovenia) and Oshee waters (Oshee Polska Sp. z o.o., Kraków, Poland), 5 juices, including Babylove (DM drogerie markt d.o.o., Berlin, Germany), Frutek (Fructal d.o.o., Ajdovščina, Slovenia), HiPP (HiPP GmbH & Co. Vertrieb KG, Pfaffenhofen an der Ilm, Germany), Yippy (Rauch Fruchtsäfte GmbH & Co OG, Vorarlberg, Austria), and Dana (Dana, d.o.o., Mirna, Slovenia), 12 energy drinks, including Flying Power (H-west B.V., Utrecht, The Netherlands), Red Bull (Red Bull GmbH, Fuschl am See, Austria), Shark (Osotspa Public Company Limited, Bangkok, Thailand), Hell (Hell Energy, Szikszó, Hungary), Burn (Coca-Cola HBC, Warsaw, Poland), Energy drink (Egger Getränke GmbH & Co OG, St. Pölten, Austria), Kong (Lidl Slovenia d.o.o k.d., Komenda, Slovenia), Q (Petrol d.d., Ljubljana, Slovenia), and Double Force (Fluidi sh.p.k. 2, Gjilan, Kosovo), and 4 instant drinks, including Caotina (Wander AG, Neuenegg, Switzerland), Nesquik (Nestlé, Vevey, Switzerland), BenQuick (Mercator-Emba Proizvodnja Hrane, d.o.o., Logatec, Slovenia), and Cedevita (Atlantic Cedevita d.o.o, Zagreb, Croatia). An overview of the tested products, indicating the labeled vitamins and their contents, is provided in Supplementary Materials (S4., entitled ‘Overview of the tested vitamin-fortified beverages’).

### 2.4. Analysis of the Commercial Products

The tested waters and energy drinks were sonicated for 5 min (Bandelin Sonorex™ ultrasonic bath, Bandelin Electronic, Berlin, Germany) and transferred into a vial for direct injection into the chromatographic system and analysis. The tested juices were hand-mixed, sonicated (5 min), and centrifuged for 10 min at 20 °C and 13,600× *g* (Centrifuge 5425 R, Eppendorf, Hamburg, Germany). The clear supernatant was filtered through a 0.45 µm Minisart^®^ RC filter (Sartorius, Göttingen, Germany) before analysis. The sample preparation of instant drinks was performed based on the manufacturer’s instructions for their everyday use (using the recommended quantity of the instant drink and solvent volume). Thus, appropriately weighted amounts of the powders were quantitatively transferred into a flask, which was filled up to the mark with 1 mM EDTA in Milli-Q water. The samples were properly homogenized with a magnetic stirrer (10 min) and/or sonicated (5 min) and filtered through a 0.45 µm RC filter before analysis.

All VFNABs were analyzed in triplicate immediately after opening. During the analysis, all tested VFNABs were within their expiration dates. Representative HPLC and LC-MS/MS chromatograms of VFNABs analysis may be found in Supplementary Materials (S5., entitled ‘Representative HPLC and LC-MS/MS chromatograms’). The contents of vitamins in the analyzed VFNABs were determined by regression analysis based on vitamin calibration standards. Within the figures and tables in this article, vitamin marks are used due to space restrictions and encompass B1—thiamin; B2—both forms, converted and presented as riboflavin; B3—niacin; B5—pantothenic acid; B6—vitamin B6 (pyridoxine); B7—biotin; B9—folic acid; B12—cyanocobalamin; C—ascorbic acid; D—cholecalciferol; and E—both forms, converted and presented as alpha-tocopherol. The representation of the determined vitamin contents within the article is based on the average of three measurements along with the standard deviation.

## 3. Results

### 3.1. Analytical Methodology

VFNABs containing water-soluble vitamins were analyzed according to a validated and published stability-indicating HPLC-DAD method for the simultaneous analysis of all main water-soluble vitamins [16]. Fat-soluble vitamins in the tested VFNABs were analyzed according to a published, validated HPLC-UV method for their simultaneous analysis [22]. VFNABs containing biotin, folic acid, cyanocobalamin, cholecalciferol, and vitamin E (as alpha-tocopherol or alpha-tocopherol acetate) at concentrations near or below the quantitation limits of the two above-described HPLC methods were additionally analyzed by LC-MS/MS methods, which were properly validated in accordance with the ICH Q2(R2) guidelines [29]. The linearity of the methods was confirmed based on the high correlation coefficients (R^2^ > 0.9997, Table S3) over the concentration ranges, which cover the expected range of concentrations, based on both the declared concentrations on the label and the results of the analysis using the HPLC methods. The DL and QL were determined based on the calibration curves (Table S3) and show the sensitivity of the methods to be sufficient for the evaluation of biotin, folic acid, cyanocobalamin, cholecalciferol, and vitamin E in the tested VFNABs (the QL was between 6- and 120-fold below the lowest declared concentration of the individual vitamin). The results obtained for intra-day and inter-day accuracy, repeatability, and intermediate precision (Table S3) are all within the acceptance criterion (±5%). All QC samples and tested VFNABs remained stable (100 ± 5%) after 24 h of storage at 8 °C.

### 3.2. Assessment of the Vitamin Labeling in the Tested Products

#### 3.2.1. Labeling of Vitamin Forms

The quality control of the tested VFNABs was initiated by the determination of the vitamin form contained and its comparison with the labeled vitamin (form). An overview of the nutrition declarations revealed that all evaluated VFNABs were labeled with generic vitamin names: thiamin, riboflavin, niacin, pantothenic acid, folic acid, biotin, vitamin B12, vitamin B6, vitamin C, vitamin D3, and vitamin E. However, only one of the tested VFNABs (an instant drink) specified the contained vitamin forms. Based on the HPLC and LC-MS/MS analyses performed, we determined that the evaluated VFNABs contained the following vitamin forms: thiamin, riboflavin and riboflavin -5-phosphate, nicotinamide, pantothenate, pyridoxine, biotin, folic acid (pteroylmonoglutamic acid), cyanocobalamin, ascorbic acid, cholecalciferol, alpha-tocopherol, and alpha-tocopheryl acetate. The determined vitamin forms corresponded to the labeled ones in the only tested VFNAB with specified vitamin forms.

#### 3.2.2. The Assessment of the Declarations and Conversion of Vitamin Amount into Their Nutrient Reference Values (NRVs)

The nutritional declarations of all evaluated VFNABs included the vitamin amounts, given in mass units (grams (g) or micrograms (µg)) per 100 g or 100 mL, the corresponding nutritional values, and a percent (%) of nutrient reference values (NRVs) per 100 g or per 100 mL. This is in accordance with EU regulations [30]. In addition to the form of expression per 100 g or 100 mL, the nutrition declarations of two of the evaluated instant drinks provided data on the nutritional values per portion (13.5 g and 15 g). This can also be considered appropriate since the portion or the unit is quantified on the label and the number of portions or units contained in the package is stated, as defined in Article 33 of the EU regulations [30]. Furthermore, we evaluated the correlation between the labeled vitamin amount and its % NRV, considering the reference vitamin intakes, as stated in point 1 of Part A of Annex XIII of the EU regulations [30]. The labeled NRV adequately reflected the labeled vitamin amounts, with two exceptions. One juice, specifically marketed for children, was labeled with 30 mg of vitamin C and 120% NRV. Since 30 mg of vitamin C corresponds to 31% NRV, we presume that the labeled NRV is adjusted to children’s needs; however, this was not specified on the label. Additionally, one of the tested vitamin waters was labeled as having 1.65 mg of thiamin (which corresponds to 150% NRV), while stating 15% NRV, which was probably a result of a conversion error in the calculation or a typographical error.

#### 3.2.3. Presence of Vitamins in Significant Amounts (Limit Amount for Their Claiming on the Label)

**Figure 1:**
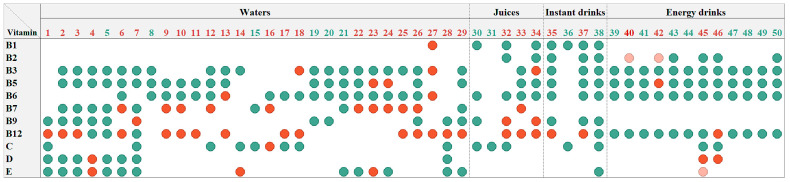
The representation of the determined vitamin amounts in the tested vitamin-fortified beverages (VFNABs) in relation to their significant amounts, as defined in the text above. Green circles represent the presence of the labeled vitamin in significant amounts, pink circles represent the presence of the unlabeled vitamin in significant amounts, and red circles represent the absence of the labeled vitamin or its presence in amounts below the significant amounts. The numerical designation of the tested VFNAB is green if it is compliant with EU legislation (the presence of all labeled vitamins in significant amounts) and red if it is not compliant (the absence or presence of at least one of the labeled vitamins in amounts below significant amounts or the presence of an unlabeled vitamin in significant amounts).

According to EU regulations, the nutrition declaration may be supplemented with an indication of the amounts of vitamins in cases of their presence in the food in at least a significant amount, which is defined in Annex XIII to Regulation (EU) No. 1169/2011 as follows: “*15% of the NRV supplied by 100 g or 100 mL in the case of products other than beverages, 7.5% of the NRV supplied by 100 mL in the case of beverages, or, 15% of the NRV per portion if the package contains only a single portion*” [30]. Therefore, when claiming the presence of a vitamin, the product must contain it in at least a significant amount. According to the nutrition declarations, all evaluated VFNABs were labeled as containing the specified vitamins in significant amounts (≥7.5% of the NRV per 100 mL of all tested VFNABs and ≥ 15% of the NRV per 100 g of the instant drinks No. 35, 36, and 37; instant drink 38 is a ready-to-drink beverage, for which the threshold 7.5% of the NRV is applicable). However, the quantitative analysis revealed that one-fifth (21%) of all evaluated vitamin contents were below the thresholds for significant vitamin amounts (Figure 1). Most of these errors were on account of biotin (4.5%) and vitamin B12 (8.6%). Additionally, three of the tested VFNABs (all three were energy drinks) were found to contain a certain vitamin (riboflavin and vitamin E) in significant amounts (7.9%, 13.8%, and 9.0% NRV). This information was not included in the nutrition declaration (Figure 1). The summarized results reveal the improper indication of at least one of the labeled vitamins in the declaration for 32 (64%) of the 50 tested VFNABs (Figure 1).

### 3.3. Determination of the Content of Vitamins in Relation to the Label Claims

**Figure 2:**
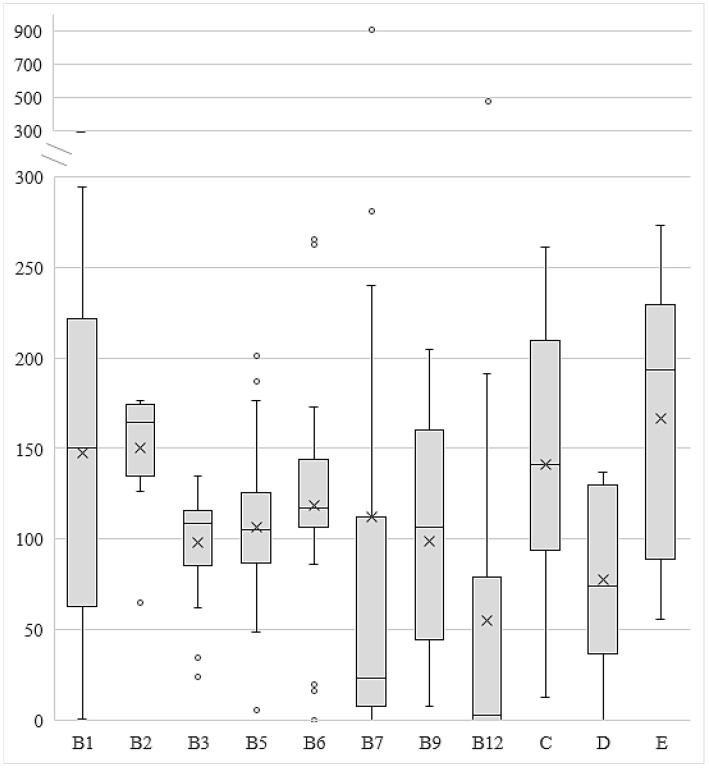
Distributions of the analytically determined vitamin contents, expressed as a percent (%) of the labeled content in the tested VFNABs. The box plots represent the 10th (lower whisker), 25th (box bottom boundary), 50th (median), 75th (box top boundary), and 90th (upper whisker) percentiles, with the outlier points lying below the lower whisker and above the upper whisker and the mean (X). Observations: thiamin, *n* = 8; riboflavin, *n* = 9; nicotinamide, *n* = 38; pantothenic acid, *n* = 37; vitamin B6, *n* = 38; biotin, *n* = 19; folic acid, *n* = 17; cyanocobalamin, *n* = 36; vitamin C, *n* = 16; vitamin D, *n* = 10; vitamin E, *n* = 15.

The content-related quality control of the VFNABs was performed by comparing the analytically determined vitamin content with its label claim. The complete set of data for all vitamins is graphed in a box plot (Figure 2). As can be seen in Figure 2, the determined contents in relation to the label claim showed different variabilities for individual vitamins. In general, the labeled content best described the actual content in the case of nicotinamide, with a mean content of 98.2% of the labeled claim and an interquartile range (IQR), calculated as the range between the first and third quartile, within 87.8 and 115.1% of the label claim, followed by pantothenic acid (a mean content of 106.4% of the label claim and an IQR between 87.6% and 124.0%), and vitamin B6 (a mean content of 118.7% and an IQR within 106.4–142.1%). The mean determined contents for biotin and folic acid were very close to the labeled claim (100 ± 2%); however, the determined contents of these two vitamins showed much higher variability (IQR within 7.7–107.5% for biotin and within 47.1–159.7% for folic acid). In the case of biotin, we observed the biggest discrepancy between the determined and labeled content in one of the tested vitamin-fortified waters, as the determined content in this product exceeded the label claim 9-fold (907.3% of the label claim). Moreover, an extremely high outlier (474.0% of the label claim) was also found for cyanocobalamin in another vitamin-fortified water. The addition of substantial overages was also evident in the case of thiamin, riboflavin, vitamin C, and vitamin E as the mean determined contents for these vitamins ranged between 141.4% and 166.2% of the label claim and as the IQR was within 143.5–173.4% for riboflavin and within 93.4–222.0% for thiamin, vitamin C, and vitamin E. On the contrary, the distribution of the determined vitamin D and cyanocobalamin contents showed mean determined contents well below the label claims (78.8% for vitamin D (IQR within 37.1–124%) and 54.9% for cyanocobalamin (IQR within 0–77.6%).

**Figure 3:**
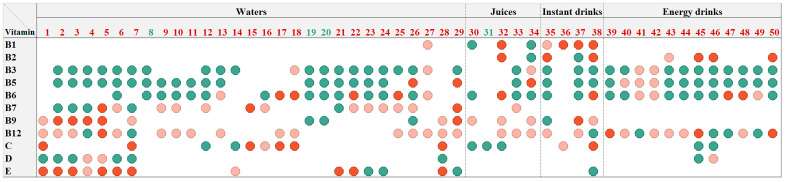
The representation of the determined vitamin content in relation to the label claim in the tested VFNABs. Green circles represent the determined vitamin content within the tolerance interval (65–150% of the label claim), pink circles represent the determined vitamin content below the lower tolerance (65% of the label claim), and red circles represent determined vitamin content above the upper tolerance (150% of the label claim). The numerical designation of the tested VFNAB is green if compliant with the EU legislation (all determined vitamin contents within the tolerance interval) and red if not compliant (content of at least one vitamin outside the tolerance interval).

The analytically determined vitamin contents in the evaluated VFNABs in relation to their label claims, were further processed in regard to EU regulations [30]. A mutual agreement between the EU Commission services and representatives of the Member States in 2012 resulted in the creation of a guidance document to help competent authorities control compliance with EU legislation, which sets up tolerances for the nutrient values declared on a label [31]. In accordance with this document, the tolerances for vitamins in foods other than food supplements range from −35% to +50% of the label claim, including measurement uncertainty; for vitamin C in liquids, higher upper tolerance values can be accepted [31]. Applying these tolerances, the results obtained for vitamin contents in all tested VFNABs were appropriately categorized into one of the three categories (below, within, and above the tolerance interval) and these are summarized in Figure 3.

In summary, 244 vitamin contents were analytically determined in VFNABs, and only half of them (51.6%) were within the tolerance interval set by the EC [31]; 20.5% were above the upper tolerance limit; and 27.9% were below the lower tolerance limit. Almost 10% (9.8%) of the labeled vitamins were below the lower limits (DL and QL) of the above-described HPLC and LC-MS/MS methods. On the other hand, 8.6% of the determined vitamin contents exceeded the label claims by more than 2-fold, which ranged up to 9-fold. Focusing on the individual product, contents outside the tolerance interval for at least one vitamin were found in the majority (92%) of the tested VFNABs, resulting in incompliance with the EU legislation.

**Figure 4:**
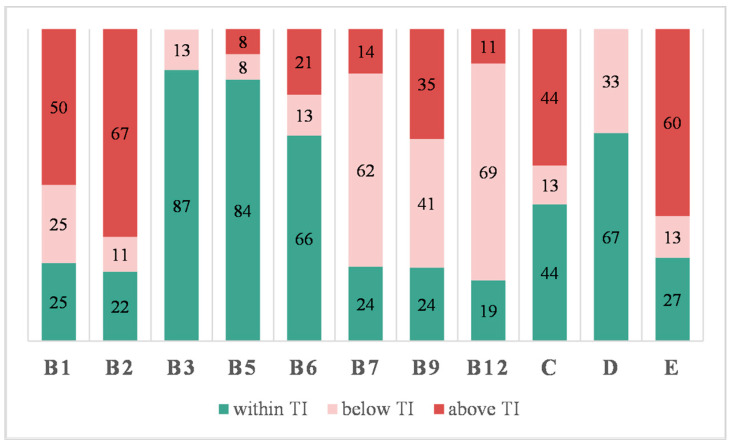
The representation of the shares (%) of tested VFNABs with determined contents of the individual vitamin below (pink), within (green), and above (red) the acceptable tolerance interval (TI) (65–150% of the label claim). Observations: thiamin, *n* = 8; riboflavin, *n* = 9; nicotinamide, *n* = 38; pantothenic acid, *n* = 37; vitamin B6, *n* = 38; biotin, *n* = 21; folic acid, *n* = 17; cyanocobalamin, *n* = 36; vitamin C, *n* = 16; vitamin D, *n* = 10; vitamin E, *n* = 15.

Further on, we explored possible associations and the causes of the determined inconsistencies in the quality of the evaluated VFNABs. Considering the dispersion of results throughout the whole range of products, a straightforward correlation between the product type (waters, juices, instant drinks, and energy drinks) or the manufacturer (several products from the same manufacturer) and the content-related quality of the products could not be established. However, differences in the determined contents in relation to the label claims are noticeable for the individual vitamins, which is detailed in Figure 4. The determined contents of niacin, pantothenic acid, vitamin D, and vitamin B6 were within the tolerance interval more often than the above-mentioned overall average (51.6%), whereas the determined thiamin, riboflavin, biotin, folic acid, cyanocobalamin, and vitamin E contents were most frequently beyond the tolerance interval (in 73–81% of the tested VFNABs).

### 3.4. Assessment of Vitamin Intake by Consumption of the Tested Products

**Figure 5:**
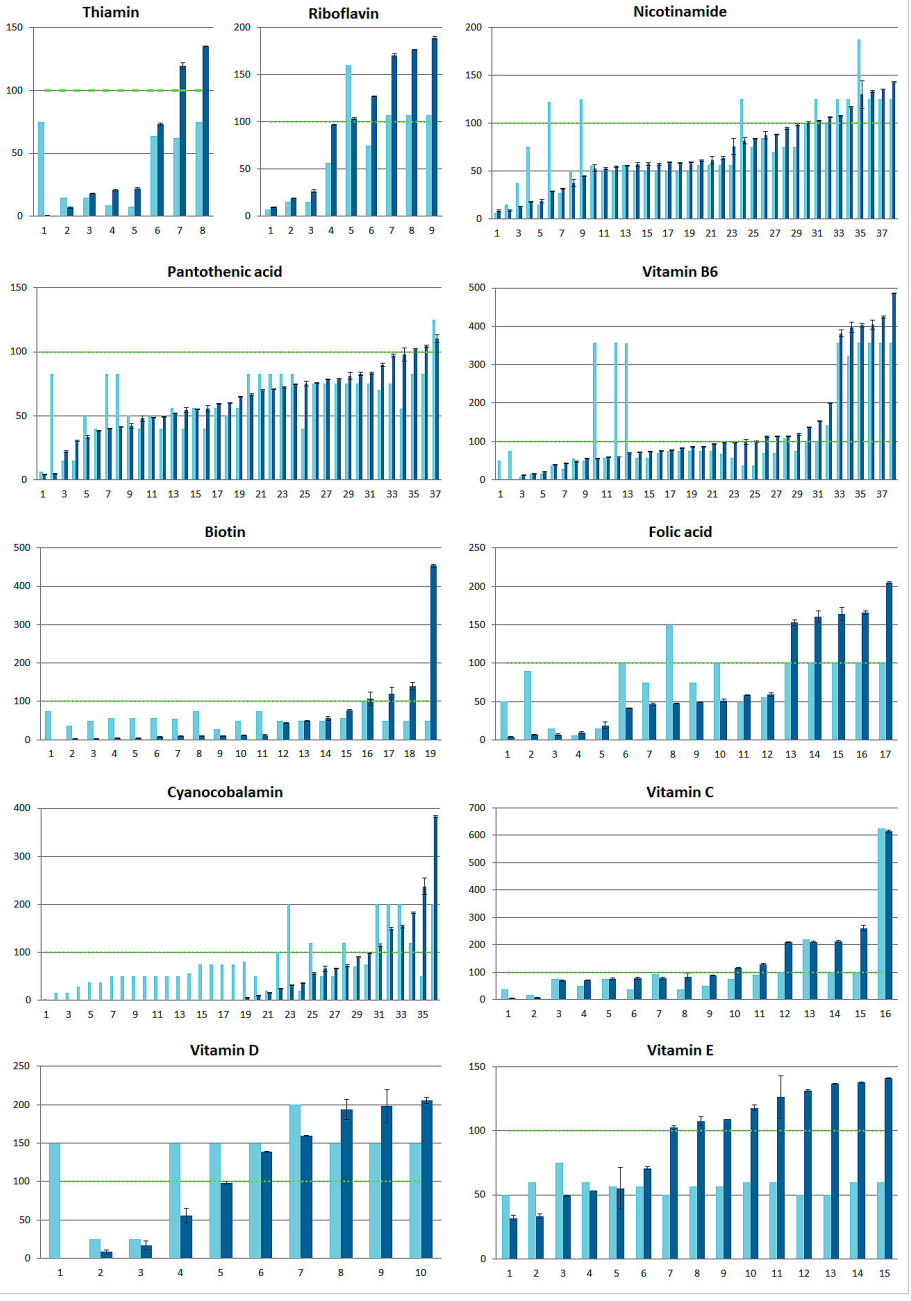
The representation of the labeled (light blues) and determined (dark blue) vitamin content (average of three measurements along with the standard deviation) in one portion of the tested VFNABs, presented as a percent (%) of the individual vitamin’s nutrient reference values (NRVs), with 100% indicated by the green dashed line.

The results of the performed content-related quality control were further used to assess vitamin intake via the consumption of the tested VFNABs. Therefore, we calculated both the labeled and analytically determined % NRV of the individual vitamin (based on the determined vitamin contents) in one portion of VFNABs. Thus, we defined one portion as the amount of the tested VFNAB recommended on the packaging or used the total packaging size in cases the portion was not specified on the label. The results for the individual vitamins in the tested VFNABs, ordered by their determined content, are graphed in Figure 5.

In terms of NRVs, the determined vitamin amounts per portion of the tested VFNABs ranged between 0 and 615.7%. In general, a majority of the determined vitamin amounts were below their NRV (Figure 5). Approximately one-third (32.9%) of all determined vitamin amounts were near or above the NRV (≥90% of the NRV). Values significantly exceeded the NRV for all vitamins, except pantothenic acid, and this was most pronounced in the case of riboflavin, vitamin B6, folic acid, and vitamins C, D, and E. In total, 32 of the 50 tested VFNABs (64%) contained an amount near or above the NRV (≥90% of the NRV) of at least one vitamin. Overall, 26 of the tested VFNABs contained more than two-fold the NRVs for vitamin B6, biotin, folic acid, cyanocobalamin, and vitamins C, D or E; 16 of the tested VFNABs contained more than three-fold the NRVs for vitamin B6, biotin, and vitamins C; and 12 of the tested VFNABs contained more than four-fold the NRVs for vitamin B6, biotin, or vitamin C, with a 616% NRV reached for vitamin C. Most of the determined daily vitamin amounts per portion of the tested VFNABs were below the maximum daily doses specified by the National Agency for Medicinal Products and Medical Devices of the Republic of Slovenia (JAZMP) for adults (above 14 years), except for two determined daily vitamin doses (for folic acid and vitamin C), which were at the limit for maximum daily doses [32]. All the determined vitamin contents were below their tolerable upper intake level (UL) for adults, which is specified by the EFSA [33].

#### Appropriateness of the Tested Products to Be Consumed by Children

**Figure 6:**
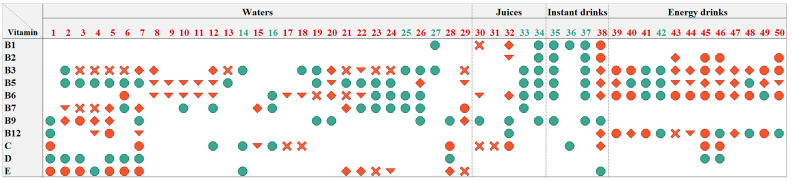
The representation of the determined vitamin content in one portion of the tested VFNABs in relation to the daily vitamin doses for children, which should not be exceeded in products other than medicines. Red circles represent the determined daily dose above the highest tolerable daily dose for children between 11 and 14 years, a red rhombus represents the determined daily dose above the highest tolerable daily dose for children between 7 and 10 years, a red triangle represents the determined daily dose above the highest tolerable daily dose for children between 4 and 6 years, a red sign x represents the determined daily dose above the highest tolerable daily dose for children between 1 and 3 years, and green circles represent the determined daily dose below the highest tolerable daily dose for children between 1 and 3 years. The numerical designation of the tested VFNAB is green if the determined daily dose of all contained vitamins is below the highest tolerable daily dose for children in all age groups and red if any of the determined daily vitamin dose exceedes their highest tolerable daily dose.

The determined vitamin amounts per portion of the tested VFNABs were further used to evaluate the appropriateness of these doses for children. Thus, we considered the significant determined contents (above the QL of the analytical methods) and compared them with the specified daily vitamin doses for each of the age groups (11–14 years; 7–10 years; 4–6 years, and 1–3 years), which should not be exceeded in products other than medicines, as specified by the JAZMP (Annex 1 of the guidelines) [32]. This assessment revealed that 20 of the 50 tested VFNABs (40%) contained at least one vitamin in amounts which exceed the highest tolerable daily dose for children in the oldest age group (between 11 and 14 years). Since the specified daily vitamin doses increases with age, exceeding the limit for children of an older age group implies it was exceeded for children in younger age groups as well. Thus, more than half (56%) of the determined vitamin amounts in the tested VFNABs exceeded the highest specified daily vitamin doses in some of the children’s age groups. Based on the summarized results (Figure 6), it can be concluded that most of the tested VFNABs contain at least one vitamin in an amount that exceeds the highest specified daily dose for children, and only 10 of the 50 tested VFNABs contained vitamins in acceptable doses for children. However, the VFNABs, which are specifically targeted and consumed by children (juices and instant drinks with numerical designations 31 and 33–37 in Figure 6), were all recognized as appropriate for consumption by children in all age groups, except juice No. 31.

## 4. Discussion

The main aim of this study was to evaluate the content-related quality of a representative sample of non-alcoholic beverages, fortified with water- and fat-soluble vitamins, on the Slovenian market. Despite the expansion of these freely accessible products on our market in recent years and the quality and safety issues associated with their widespread use, published data on their quality are quite scarce. Therefore, this study, which is so far the first published comprehensive evaluation of a broad sample of VFNABs in regard to vitamin content, provides relevant data on their quality. These data are not only relevant to the scientific community but also to both consumers and policymakers. An appropriate, specific, accurate, and precise analytical method, with proper sensitivity for the quantitative determination of low vitamin contents (i.e., a few µg/L of vitamin B12, D, and biotin in some VFNABs), is a prerequisite for quality control. For such purposes, we utilized two validated and previously published HPLC methods for the analysis of water-soluble and fat-soluble vitamins. These were additionally complemented by LC-MS/MS methods, used in the analysis of vitamin concentrations below the lower limits (DL and QL), and we performed result verification. The utilized LC-MS/MS methods were validated in accordance with the ICH Q2(R2) guidelines [29]. The results obtained based on the calibration curves and quality control samples with concentrations adjusted to the levels of the individual vitamin contents in the VFNABs (Tables S2 and S3) prove that the utilized method provides proper linearity, sensitivity, accuracy, and repeatability and is therefore applicable for their evaluation in VFNABs.

The quality control of the tested VFNABs was performed in regard to the applicable European and national (Slovenian) legislation. The regulatory framework on the addition of vitamins to foods in the EU is covered by Regulation (EC) 1925/2006 [34] and its amendments. Regulation (EU) No. 1169/2011 of the European Parliament and of the Council, passed on 25 October 2011, lays down the rules on the provision of food information to consumers. In accordance with this regulation, the mandatory nutrition declaration includes the energy values and the amounts of fat, saturates, carbohydrates, sugars, protein, and salt and may be supplemented with an indication of the amounts of one or more of the vitamins, listed in Annex XIII, if present in significant amounts as defined in Annex XIII [30]. Evaluating the correctness of the labeling, we focused on both of the aspects covered by this regulation: the contained vitamin form and its presence in significant amounts. The specific vitamins contained in all tested VFNABs (Section 3.2.1, Labeling of vitamin forms) were given as a list of vitamins and their forms that can be added to foods, as specified in point 1 of Annex XIII of the EU regulation [34]. However, it was noted that manufacturers are typically not specific when labeling the contained vitamins, as they are typically labeled with the generic terms. Only 1 of the 50 tested VFNABs specified the contained vitamin forms, which is not surprising as the specification of the contained vitamins’ form is not mandatory for foods. Moreover, although the labeled vitamin amounts were mainly properly converted into their nutritional values (% NRV on the nutrition declarations) (Section 3.2.2, Assessment of the declarations and conversion of vitamin amount into their nutrient reference values (NRVs)), the determination of the actual vitamin content revealed several inadequacies. Namely, a majority (31) of the nutrition declarations regarding the 50 tested VFNABs (Figure 1) were improperly supplemented with an indication of the amounts of the vitamins, since their presence in the VFNABs was below the levels that constitute significant amounts, as defined in Annex XIII of Regulation (EU) No. 1169/2011 [30]. Three additional VFNABs contained a certain vitamin (riboflavin or vitamin E) in significant amounts, as defined in Annex XIII of Regulation (EU) No. 1169/2011 [30], but were not included in the nutrition declaration (Figure 1).

The perceived trends of inadequate VFNAB labeling and quality were further investigated by comparing the analytically determined vitamin contents, as set by the EC [31], with their label claims, which were outside the tolerance interval of 65–150% claimed by the label in almost half (48.4%) of the 244 assessed vitamins (Figure 3). Since contents outside the tolerance interval were determined in almost all tested VFNABs (92% of the 50 tested VFNABs) and in all 4 VFNAB types (waters, juices, energy drinks, and instant drinks), a direct correlation between the product type or the manufacturer and the content-related quality of the products could not be established. Instead, differences in the determined contents in relation to the label claims were found to be vitamin-specific (Figure 2, Figure 3 and Figure 4). Thus, the determined contents of nicotinamide, followed by pantothenic acid, and vitamin B6, showed the best correlation with and lowest variability in relation to the label claims. The high variability of the results and contents outside the tolerance interval, ranging between 0% and 907% of the label claim for the remaining vitamins (Figure 2), is a demonstration of the improper formulation and consequently also improper quality of the tested VFNABs. The inappropriate quality shown may be a consequence of an intentional (e.g., the absence of ingredients or the use of ingredients of lower quality) or unintentional act (inappropriate manufacture, formulation, and stabilization). Therefore, during the design and manufacture of VFNABs, manufacturers should take into consideration the fact that vitamin degradation may be caused by both external (e.g., temperature, light, and exposure to oxidative conditions) and internal factors (e.g., medium, presence of other vitamins, and constituents) [16,17,18,20,22,35] and ensure their proper stabilization. In that sense, vitamin contents being below the label claims, most typical for cyanocobalamin (IQR within 0–77.6%) and vitamin D (IQR within 37.1–124%), may be a consequence of vitamin instability in aqueous solutions, which is well described in the scientific literature [20,35,36,37]. Additionally, as VFNABs commonly contain several different vitamins, there is a possibility of their interaction, which can also affect the stability of individual vitamins and reduce or increase the overall stability of the product. Typical examples are the reduced cyanocobalamin stability seen in the presence of ascorbic acid, riboflavin, thiamin, and nicotinamide; the reduced stability of thiamin, folic acid, and ascorbic acid seen in the presence of riboflavin; the reduced stability of folic acid seen in the presence of thiamin; and the increased stability of vitamins susceptible to oxidation (biotin, folic acid, vitamin E and ascorbic acid) seen in the presence of ascorbic acid and/or vitamin E, which act as (synergistic) antioxidants [35,38,39,40,41,42]. In this study, we also observed that VFNAB manufacturers also practice the addition of overages (the addition of higher vitamin amounts than labeled), which is a common approach for the compensation of vitamin losses during the manufacture and storage of food supplements and medicines [20,21,23,35,43]. Thus, we noted the use of non-standardized overages, both in different products from the same manufacturer and by different manufacturers. The addition of overages was detected for all vitamins; however, the most common and highest overages were observed in the case of thiamin, riboflavin, vitamin C, and vitamin E (Figure 2 and Figure 4), which are unstable vitamins and also participate in various vitamin–vitamin interactions. However, in our judgment, the addition of such high amounts, which exceed the labeled claims by several folds and considerably exceed their expected degradation during manufacture and storage, is not justified in regard to the safety of these products, especially since they are used by practically all population subgroups. As reported by Flynn et al., a 25% overage of vitamins B6, D, and E, folic acid, and retinol in fortified foods and supplements can be considered safe for both children and adults [44]. Nonetheless, the long-term consumption of VFNABs with higher vitamin contents than declared may result in their excessive intake with serious health consequences. This is especially true for fat-soluble vitamins, which are excreted from the body less efficiently than water-soluble vitamins [23,32,45,46]. Therefore, the performed content-related quality control was further utilized as a foundation for the accurate evaluation of individual vitamin intake by the ingestion of one portion of the tested VFNABs and the performance of an overall assessment of the tested VFNABs as vitamin sources. Based on the results obtained (Figure 5), it can be concluded that the tested VFNABs represent a significant source of vitamins that can fully meet daily needs and in several cases even considerably exceed them (up to 616% of the NRV for vitamin C). All determined vitamin contents are below their tolerable upper intake level (UL) for adults, defined as the maximum level of total chronic intake of a nutrient from all sources judged to be unlikely to pose the risk of adverse health effects in humans [33]. However, such high vitamin amounts represent a concerning safety issue, especially since these products are typically used in combination with other vitamin sources (food and additional products—other fortified foods, food supplements, or medicines with individual or multiple vitamins) and in all age groups. In the absence of uniform European guidelines regarding the maximum allowed vitamin amounts in food products, we rely on national guidelines in the further evaluation of such high-dose VFNABs. The JAZMP, which is the competent authority for medicines and medicinal devices for human and veterinary use in Slovenia, has published guidelines for the determination of products, for which there is doubt as to whether they are classified as medicinal products or as belonging to another product group, such as medical devices; foods, including novel foods, food supplements, and foods for special health purposes; or cosmetic products, which are subjected to other regulations. According to these guidelines, although the human body’s homeostatic mechanisms of absorption, metabolism, and elimination protect against high doses, especially of water-soluble vitamins, burdening the organism with higher vitamin doses is not scientifically justified, especially as positive effects of higher vitamin doses on human health are often not established and also due to possible safety issues associated with their long-term consumption. Therefore, these guidelines specify the daily vitamin doses which, from the point of view of public health protection, should not be exceeded in products other than medicines (Annex 1 of the guidelines) [32]. Generally, the maximum daily doses are set up to five times their NRVs for water-soluble vitamins, and up to two-fold their NRVs for fat-soluble vitamins, and are below their tolerable upper intake levels established by the Scientific Committee on Nutrition, Novel Foods and Allergens at the European Food Safety Agency (EFSA) [33]. In general, the determined daily vitamin doses per portion of the tested VFNABs were below the maximum daily doses for those over 14 years old. Two of the determined vitamin daily doses (for folic acid and vitamin C) were at or slightly above their limit for maximum daily doses, as specified by the JAZMP [32].

Some of these products are specifically targeted and consumed by children (e.g., juices and instant drinks), who generally have lower vitamin requirements compared with adults. Therefore, we were additionally interested in the suitability of the tested VFNABs designated for consumption by children from a safety point of view. Thus, we compared the determined vitamin amounts in the tested VFNABs with the daily vitamin doses for each of the age groups (1–3 years; 4–6 years; 7–10 years; and 11–14 years), which should not be exceeded in products other than medicines, as specified by the JAZMP [45]. On this basis, we concluded that the doses of vitamins in products intended for children (some of the tested juices and instant drinks) are mostly adapted to their needs. On the other hand, since a great majority of the tested waters (86%) and energy drinks (92%) contained higher amounts of at least one vitamin than specified by the JAZMP regarding some of the children’s age groups, we conclude that these VFNABs are inappropriate for use by children in terms of vitamin content. In that sense, we also address the need for more appropriate labeling of these products with the addition of a warning statement that the product is not to be consumed by children. The consumption of energy drinks by children and adolescents is not only a cause for concern in regard to excessive vitamin intake, but also because of their high caffeine, sugar, and caloric content. Because of the potential risks for cardiovascular, gastrointestinal, and mental health issues associated with energy drink consumption, we encourage their stricter regulation at both state levels and beyond, following the lead of Lithuania, which is the first European Union member state to prohibit their sale to children. Similar regulations have also been introduced by Latvia, Sweden, and certain Spanish autonomous regions, whereas Denmark, Turkey, and Uruguay have entirely prohibited their use [47].

## 5. Conclusions

The quality of vitamin-fortified beverages depends not only on the quality of the raw materials but also on the formulation and manufacturing processes, which are usually not accompanied by proper quality control and stability evaluation. In recent times, which have featured their widespread and growing use and loose regulation by the competent authorities, consumers have come to rely on the nutritional declaration achieving the desired effects. The results of the first published comprehensive study on the content-related quality control of vitamin-fortified beverages (waters, juices, instant drinks, and energy drinks) reveal several irregularities and inconsistencies including the labeling of vitamins, which are present in lower-than-significant amounts in the nutrition declaration; the absence of vitamins, which are present in higher-than-significant amounts in the nutrition declaration; vitamin contents outside the tolerance interval (65–150% of the label claim) in almost all tested VFNABs; and high variability in the determined contents (0–907% of the label claim). Therefore, the performed content-related quality control study can be utilized as a foundation and recommendation for the manufacturers in terms of committing to and pursuing the production of high-quality vitamin-fortified beverages. This includes the accurate evaluation of vitamin contents and the provision of appropriate vitamin amounts from their manufacture to the end of their shelf life. Evaluating these products as vitamin sources, we outlined that they should be considered as a significant source of vitamins since one-third of all determined vitamin amounts fully cover daily needs, and in several cases even considerably exceed them (up to 616% of the nutrient reference values). Such high vitamin amounts should be used with caution, due to possible safety issues associated with their consumption, and are not in accordance with national regulations. These safety issues are even more pronounced in children, who have lower requirements and upper tolerance safety limits. 

## Data Availability

Data are contained within the article or Supplementary Material.

## Supplementary Materials

The following supporting information can be downloaded at: https://www.mdpi.com/article/10.3390/nu16223872/s1. Table S1. Mass spectrometry parameters for the analysis of biotin (B7), folic acid (B9), cyanocobalamin (B12), cholecalciferol (D), alpha-tocopherol (E), and alpha-tocopherol acetate (E-acetate). Table S2. Concentrations of calibration standards and QC samples. Table S3. Validation data: linearity, DL, QL, intra- and inter-day accuracy, repeatability, and intermediate precision of the method. Table S4. Overview of the tested VFNABs: waters (W), juices (J), energy drinks (ED), and instant drinks (ID), indicating vitamin forms and their contents in the specified unit (100 mL for drinks and per portion for instant drinks), as labeled on the package. Figure S1. HPLC chromatogram obtained by the analysis of water-soluble vitamins in vitamin-fortified water at: (a) 210 nm—the wavelength used for the detection of vitamin B5 also representing the chromatographic separation of vitamins B3 (retention time 6.8 min) and B5 (retention time 9.5 min); and (b) 260 nm—the wavelength used for the detection of vitamin B3. Figure S2. LC-MS/MS chromatogram obtained by the analysis of water-soluble vitamins in vitamin-fortified water. The top trace represents vitamin B12 (retention time 3.5 min); the middle trace represents vitamin B9 (retention time 3.2 min) and the bottom trace represents vitamin B7 (retention time 3.1 min). Figure S3. LC-MS/MS chromatogram obtained by the analysis of fat-soluble vitamins in vitamin-fortified water. The top trace represents vitamin E, in the form of alpha-tocopherol acetate (retention time 4.8 min), and the bottom trace represents vitamin D (retention time 1.9 min). Figure S4. Representative HPLC chromatogram obtained by the analysis of water-soluble vitamins in vitamin-fortified apple juice at: (a) 210 nm—the wavelength used for the detection of vitamin B5 also representing the chromatographic separation of vitamins B6 (retention time 4.3 min), B3 (retention time 6.9 min), and B5 (retention time 9.4 min); (b) 290 nm—the wavelength used for the detection of vitamin B6; (c) 260 nm—the wavelength used for the detection of vitamin B3. Figure S5. LC-MS/MS chromatogram obtained by the analysis of water-soluble vitamins in vitamin-fortified apple juice. The top trace represents the absence of the labeled vitamin B12 (retention time 3.5 min); the middle trace represents the absence of vitamin B9 (retention time 3.2 min) and the bottom trace represents vitamin B7 (retention time 3.1 min). Figure S6. Representative HPLC chromatogram obtained by the analysis of water-soluble vitamins in vitamin-fortified energy drink at: (a) 210 nm—the wavelength used for the detection of vitamin B5 also representing the chromatographic separation of vitamins B6 (retention time 4.4 min), B3 (retention time 6.9 min), and B5 (retention time 9.4 min); (b) 290 nm—the wavelength used for the detection of vitamin B6; (c) 260 nm—the wavelength used for the detection of vitamin B3. Figure S7. LC-MS/MS chromatogram obtained by the analysis of water-soluble vitamins in vitamin-fortified energy drink. The top trace represents vitamin B12 (retention time 3.5 min); the middle trace represents the absence of vitamin B9 (retention time 3.2 min) and the bottom trace represents the absence of vitamin B7 (retention time 3.1 min).
