# Supplementary material for: Content-Related Quality Control of Water- and Fat-Soluble Vitamins in Fortified Non-Alcoholic Beverages
Source: Nutrients. 2024 Nov 13;16(22):3872. doi: 10.3390/nu16223872 (PMC11597373; doi:10.3390/nu16223872)
Supplement: Supplementary file 1 [file nutrients-16-03872-s001.zip › nutrients-3288991-supplementary.pdf]

nutrients

Supplementary materials

Article title

Content-related quality control of water- and fat-soluble vitamins in fortified non-alcoholic beverages

Authors

Žane Temova Rakuša and Robert Roškar*

University of Ljubljana, Faculty of Pharmacy, Aškerčeva cesta 7, 1000 Ljubljana, Slovenia

*Corresponding author: Tel: +386 1 4769 500, e-mail: robert.roskar@ffa.uni-lj.si

Contents

S1. LC-MS/MS analysis

S2. Preparation of samples for validation of the LC-MS/MS methods

S3. LC-MS/MS method validation

S4. Overview of the tested vitamin-fortified beverages

S5. Representative HPLC and LC-MS/MS chromatograms

S1. LC-MS/MS analysis

Table S1. Mass spectrometry parameters for the analysis of biotin (B7), folic acid (B9), cyanocobalamin (B12), cholecalciferol (D), alpha-tocopherol (E), and alpha-tocopherol acetate (E-acetate).

Vitamin	Retention time (min)	Precursor ion (m/z)	Product ion (m/z)	Fragmentor (V)	Collision energy (eV)	Polarity
B7	3.1	245.1	227.1	92	9	+
B9	3.2	442.2	295.0	92	8	+
B12	3.5	678.6	147.2	168	36	+
D	1.9	385.4	91.1	92	65	+
E	3.0	431.4	165.1	108	17	+
E-acetate	4.8	473.2	165.1	108	45	+

S2. Preparation of samples for validation of the LC-MS/MS methods

A mixture of biotin, folic acid, and cyanocobalamin as well as a mixture of alpha-tocopherol and alpha-tocopherol acetate and cholecalciferol solution were prepared fresh daily for three consecutive days of the validation. Because of the low solubility in water, 1 mM NaOH was used for the dissolution of biotin and folic acid. Cyanocobalamin stock solution was prepared in Milli-Q water. The mixture of biotin, folic acid, and cyanocobalamin was filled up to the volume of the flask and further diluted to obtain the calibration standards or quality control samples with Milli-Q water. The solvent for cholecalciferol, alpha-tocopherol, and alpha-tocopherol acetate was methanol.

Seven calibration standards were prepared from each of the two prepared mixtures (Table S2). Quality control (QC) solutions (in triplicate) were prepared in the same manner from separately prepared stock solutions at three concentration levels covering low (QC_L), medium (QC_M), and high (QC_H) concentration ranges (Table S2).

Table S2. Concentrations of calibration standards and QC samples.

		B7 (µg/L)	B9 (mg/L)	B12 (µg/L)	D (µg/L)	E (mg/L)	E-acetate (mg/L)
**Calibration standards**		10.0	0.1	2.5	2.3	2.0	2.0
		20.0	0.2	5.0	4.6	4.0	4.0
		40.0	0.4	10.0	9.2	8.0	8.0
		80.0	0.8	20.0	18.4	16.0	16.0
		120.0	1.2	30.0	27.6	24.0	24.0
		160.0	1.6	40.0	36.8	32.0	32.0
		200.0	2.0	50.0	46.0	40.0	40.0
**QC samples***	**QC_L**	30.0	0.3	7.5	6.9	6.0	6.0
	**QC_M**	100.0	1.0	25.0	23.0	20.0	20.0
	**QC_H**	150.0	1.5	37.5	34.5	30.0	30.0

*QC_L, QC_M, and QC_H – quality control samples with low, medium, and high concentrations.

S3. LC-MS/MS method validation

The LC-MS/MS methods were validated according to ICH guidelines Q2(R2) in terms of linearity, detection limit (DL), quantitation limit (QL), precision, accuracy, and sample stability [29].

Linearity was assessed with a plot of signals as a function of the evaluated vitamin concentration. Based on the responses of the seven calibration standards (Section Preparation of samples for validation of the LC-MS/MS method), a regression line by the method of least squares was calculated during three consecutive days. The acceptance criterion was $R^2 > 0.999$.

The lower range limits (DL and QL) estimation was based on the standard deviation of the y-intercepts (σ) and the slope of the lower range calibration curves. Thus, DL was calculated as DL = 3.3 σ/S and QL = 10 σ/S. DL and QL values are expressed as concentrations (µg/L).

Accuracy and precision were determined based on the three QC samples: QC_L, QC_M, and QC_H, prepared in triplicate over three consecutive days. Accuracy was determined both intra- and inter-day, as a ratio (%) of measured versus the theoretically expected concentration and was expected to be within 100 ± 5%. Precision evaluation included assessment of repeatability and intermediate precision. Both were expressed as relative standard deviation (RSD) for the analysis of the QC samples on each concentration level and were expected to be ≤ 5%. Repeatability was assessed using the nine determinations of the QC samples (three concentrations, in triplicates). Intermediate precision was assessed within the three consecutive days of the validation.

Sample stability was determined on each QC level and also in samples from all tested preparations. Sample stability was expressed as a percentage of the remaining response after 24h of storage at 8°C in relation to the initial response and was expected to be within 100 ± 5%.

Table S3. Validation data: linearity, DL, QL, intra- and inter-day accuracy, repeatability, and intermediate precision of the method.

Vitamin	Range (mg/L)*	R^2	DL (µg/L)	QL (µg/L)	Intra-/Inter-day accuracy (%)			Repeatability / Intermediate precision (%)		
					QC_L	QC_M	QC_H	QC_L	QC_M	QC_H
B7	10.0 – 200.0	0.9999	0.40	1.22	100.6 / 103.8	101.3 / 97.6	101.3 / 98.7	1.0 / 2.3	1.0 / 2.1	1.3 / 3.1
B9	0.1 – 2.0	0.9997	4.28	1.41	100.3 / 95.5	98.2 / 101.7	99.5 / 97.3	1.1 / 2.7	1.0 / 2.6	1.1 / 4.3
B12	2.5 – 50.0	0.9999	0.11	0.34	102.0 / 103.2	100.0 / 103.8	100.6 / 103.8	1.5 / 2.2	1.7 / 2.3	1.7 / 2.5
D	2.3 – 46.0	0.9997	0.28	0.86	99.7 / 101.2	97.1 / 97.0	98.0 / 102.0	0.5 / 2.7	0.8 / 1.9	0.8 / 2.0
E	2.0 – 40.0	0.9999	23.87	72.34	102.8 / 100.0	99.1 / 97.4	100.3 / 98.8	1.5 / 2.5	0.4 / 0.8	1.1 / 2.0
E-acetate	2.0 – 40.0	0.9999	25.93	78.59	100.3 / 100.2	100.2 / 101.1	100.5 / 103.5	0.6 / 1.0	0.1 / 0.9	0.6 / 1.4

* the unit for vitamin B7, vitamin B12, and vitamin D range is µg/L.

S4. Overview of the tested vitamin-fortified beverages

Table S4. Overview of the tested vitamin-fortified non-alcoholic beverages (VFNABs): waters (W), juices (J), energy drinks (ED), and instant drinks (ID), indicating vitamin forms and their contents in the specified unit (100 mL for drinks and per portion for instant drinks), as labeled on the package.

VFNABs No.	Labeled vitamin form	Labeled content	
		mg*/unit	% RDA/unit
1, 2, 3, 4 (W)	Niacin	1.6	10
	Pantothenic acid	0.48	8
	Biotin	5	10
	Folic acid	40	20
	B12	0.25	10
	D	1.5	30
	E	1.4	12
5 (W)	Niacin	1.6	10
	Pantothenic acid	0.48	8
	B6	0.28	20
	Biotin	5	10
	B12	0.25	10
	D	2	40
	E	1.4	12

VFNABs No.	Labeled vitamin form	Labeled content	
		mg*/unit	% RDA/unit
6 (W)	Niacin	3.2	20
	Pantothenic acid	0.6	10
	Biotin	10	20
	Folic acid	20	10
	B12	0.26	10
	C	16	20
	D	1.5	30
	E	1.2	10
7 (W)	Folic acid	40	20
	B12	0.25	10
	C	16	20
	D	1.5	30
	E	1.2	10
8 (W)	Niacin	2.4	15
	Pantothenic acid	0.9	15
	B6	0.21	15
9 (W)	Pantothenic acid	0.9	15
	B6	0.21	15
	Biotin	7.5	15
	B12	0.375	15
10, 11 (W)	Pantothenic acid	0.9	15
	B6	0.21	15
	B12	0.375	15
12 (W)	B3	1.6	15
	B5	0.6	15
	B6	0.14	15
	B7	5	15
	C	8	15
13 (W)	B3	1.6	15
	B5	0.6	15
	B6	0.14	15
	B12	0.25	15
14 (W)	B7	5	10
	C	8	10
15 (W)	B3	5	10
	C	8	10
16 (W)	B6	0.105	7.5
	Biotin	3.75	7.5
	C	6	7.5
17 (W)	B6	0.105	7.5
	B12	0.1875	7.5
	C	6	7.5
18 (W)	Niacin	1.2	7.5
	B6	0.105	7.5
	B12	0.188	7.5
	C	6	7.5
19, 20 (W)	Niacin	2.7	17
	Pantothenic acid	0.9	15
	B6	0.29	21

VFNABs No.	Labeled vitamin form	Labeled content	
		mg*/unit	% RDA/unit
	Folic acid	30	15
21, 22, 23, 24 (W)	Niacin	1.2	7.5
	Pantothenic acid	0.45	7.5
	B6	0.105	7.5
	Biotin	3.75	7.5
	E	0.9	7.5
25 (W)	Niacin	1.6	10
	B6	0.17	10
	Biotin	5	10
	B12	0.25	10
26 (W)	Niacin	1.6	10
	Pantothenic acid	0.6	10
	B6	0.14	10
	Biotin	5	10
	Folic acid	20	10
	B12	0.25	10
27 (W)	B1	1.65	15
	Niacin	2.4	15
	B6	0.21	15
	B12	0.375	15
28 (W)	Folic acid	40	20
	B12	0.25	10
	C	16	20
	D	1.5	30
	E	1.2	10
29 (W)	Niacin	1.6	10
	Pantothenic acid	0.48	8
	Biotin	5	10
	Folic acid	40	20
	B12	0.25	10
	E	1.44	12
30 (mixed fruit J)	Thiamin	0.35	32
	B6	0.48	34
	Folic acid	150	75
	C	37.5	47
31 (mixed fruit J)	C	30	120
32 (strawberry J)	Thiamin	0.35	31
	Riboflavin	0.4	28
	B6	0.5	35
	Folic acid	90	45
	B12	1	40
	C	37.5	46
33 (apple J)	Niacin	2.4	15
	Pantothenic acid	0.9	15
	B6	0.21	15
	Biotin	7.5	15
	B12	0.38	15
34 (apple, carrot, banana J)	B1	0.083	7.5
	B2	0.105	7.5
	B3	1.2	7.5

VFNABs No.	Labeled vitamin form	Labeled content	
		mg*/unit	% RDA/unit
	B5	0.45	7.5
	B6	0.105	7.5
	B9	15	7.5
	B12	0.188	7.5
35, 36, 37, 38 (ED)	Niacin	8	50
	Pantothenic acid	2	33
	B6	2	143
	B12	2	80
39 (ED)	Niacin	7.9	49
	Pantothenic acid	1.98	33
	B6	2	142
	B12	2	80
40 (ED)	Riboflavin	0.9	64
	Niacin	8	50
	Pantothenic acid	2	33
	B6	2	143
	B12	1.2	48
41 (ED)	Niacin	8	50
	Pantothenic acid	2	33
	B6	2	143
	B12	1.2	48
42 (ED)	Riboflavin	0.6	43
	Niacin	12	75
	Pantothenic acid	3	50
	B6	1.8	129
	B12	1.2	48
	C	70	88
	D	0.5	10
43 (ED)	Riboflavin	0.6	43
	Niacin	8	50
	Pantothenic acid	2	33
	B6	0.8	57
	B12	0.2	8
	C	200	250
	D	0.5	10
44 (ED)	Niacin	4.5	28
	Pantothenic acid	1.7	28
	B6	0.4	28
	B12	0.7	28
45 (ED)	Niacin	6.4	40
	Pantothenic acid	2	33
	B6	0.56	40
	B12	1	40
46 (ED)	Riboflavin	0.6	43
	Niacin	8	50
	Pantothenic acid	2	33
	B6	2	143
	B12	0.2	8

VFNABs No.	Labeled vitamin form	Labeled content	
		mg*/unit	% RDA/unit
47 (ID)	Thiamin	0.85	75
	Riboflavin	1.05	75
	Niacin	12	75
	Pantothenic acid	4.5	75
	B6	1.05	75
	Folic acid	150	75
	B12	1.9	75
	C	60	75
	E	9	75
48 (ID)	Thiamin	0.165	15
	Riboflavin	0.21	15
	Niacin	2.4	15
	Pantothenic acid	0.9	15
	B6	0.21	15
	Folic acid	30	15
	B12	0.375	15
49 (ID)	B1	0.102	9.24
	C	14.7	18.34
50 (ID)	B1	0.084	7.5
	B2	0.096	6.75
	Niacin	1.08	6.75
	Pantothenic acid	0.36	6
	B6	0.12	8.55
	Folic acid	12	6
	B12	0.06	2.4

* the unit for biotin, folic acid, vitamin B12, and vitamin D is μg.
The numerical designation of the tested VFNABs does not correlate with their numerical designation in Figures 1, 3 and 6.

S5. Representative HPLC and LC-MS/MS chromatograms

S5.1 Representative HPLC and LC-MS/MS chromatograms from the analysis of vitamin-fortified water with numerical designation 5 in Figures 1, 3, and 6

a)

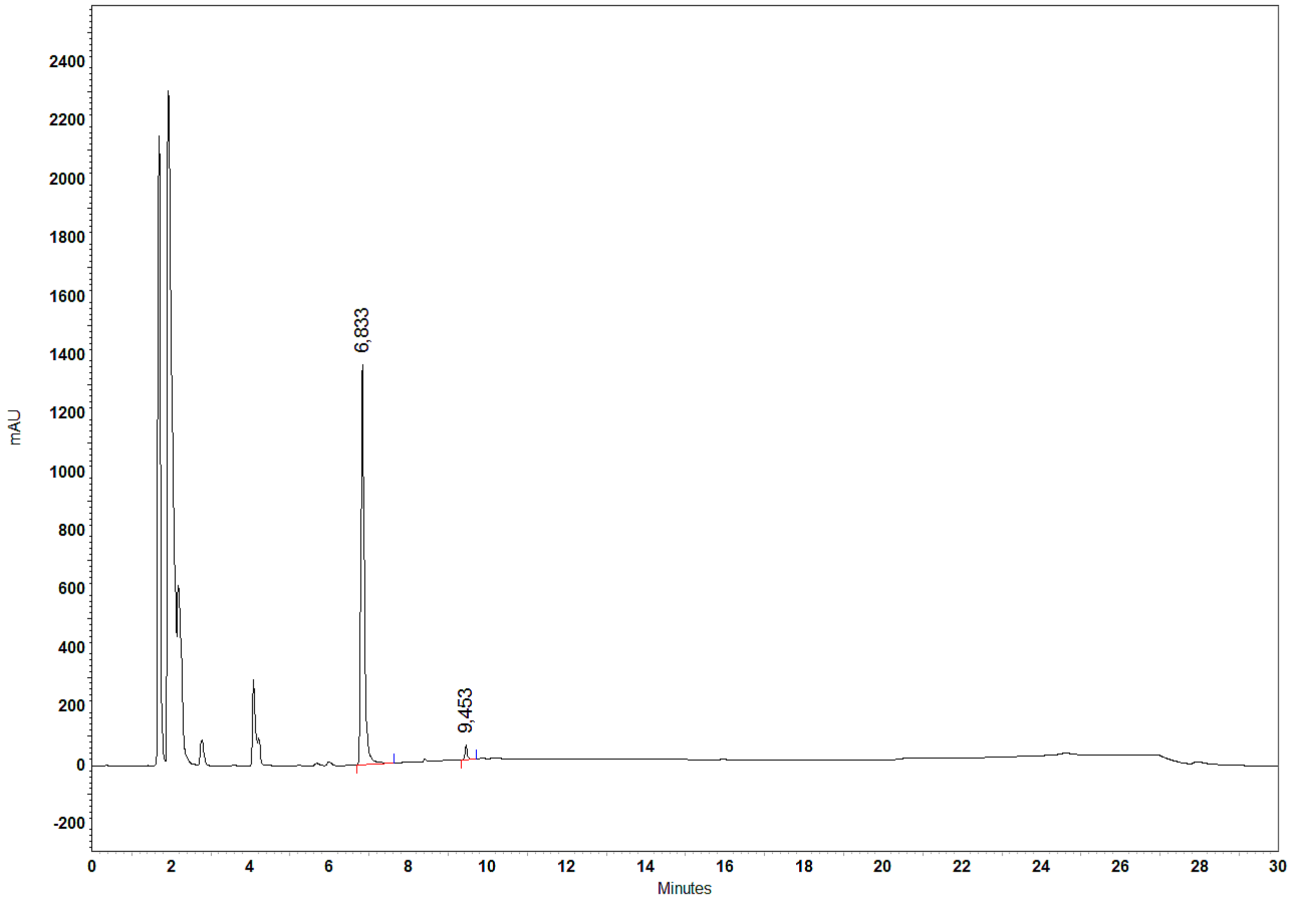

b)

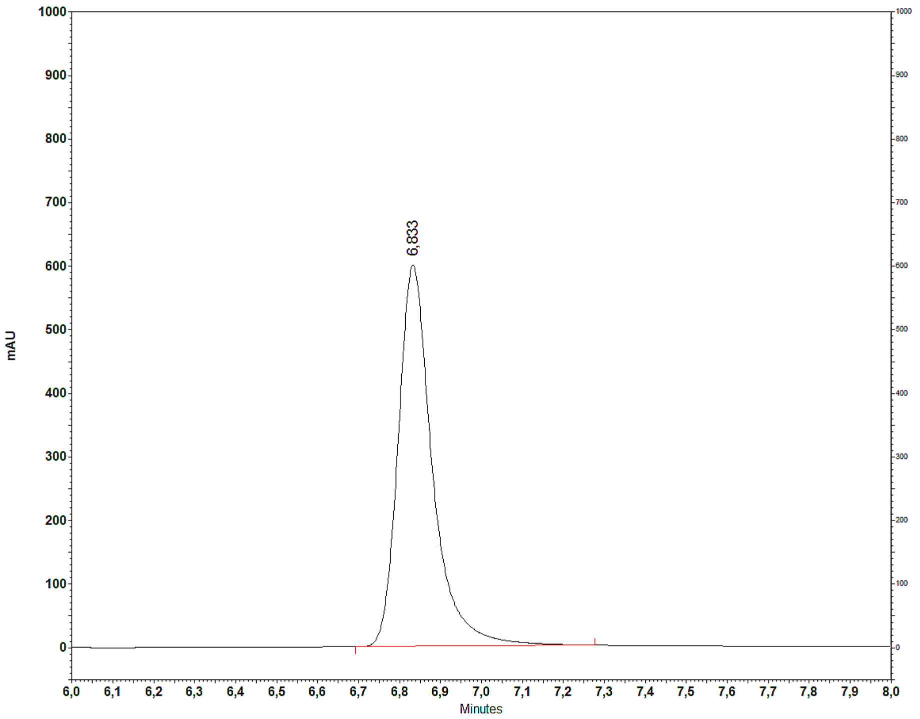

Figure S1. HPLC chromatogram obtained by the analysis of water-soluble vitamins in vitamin-fortified water at:
a) 210 nm - the wavelength used for the detection of vitamin B5 also representing the chromatographic separation of vitamins B3 (retention time 6.8 min) and B5 (retention time 9.5 min); and
b) 260 nm - the wavelength used for the detection of vitamin B3.

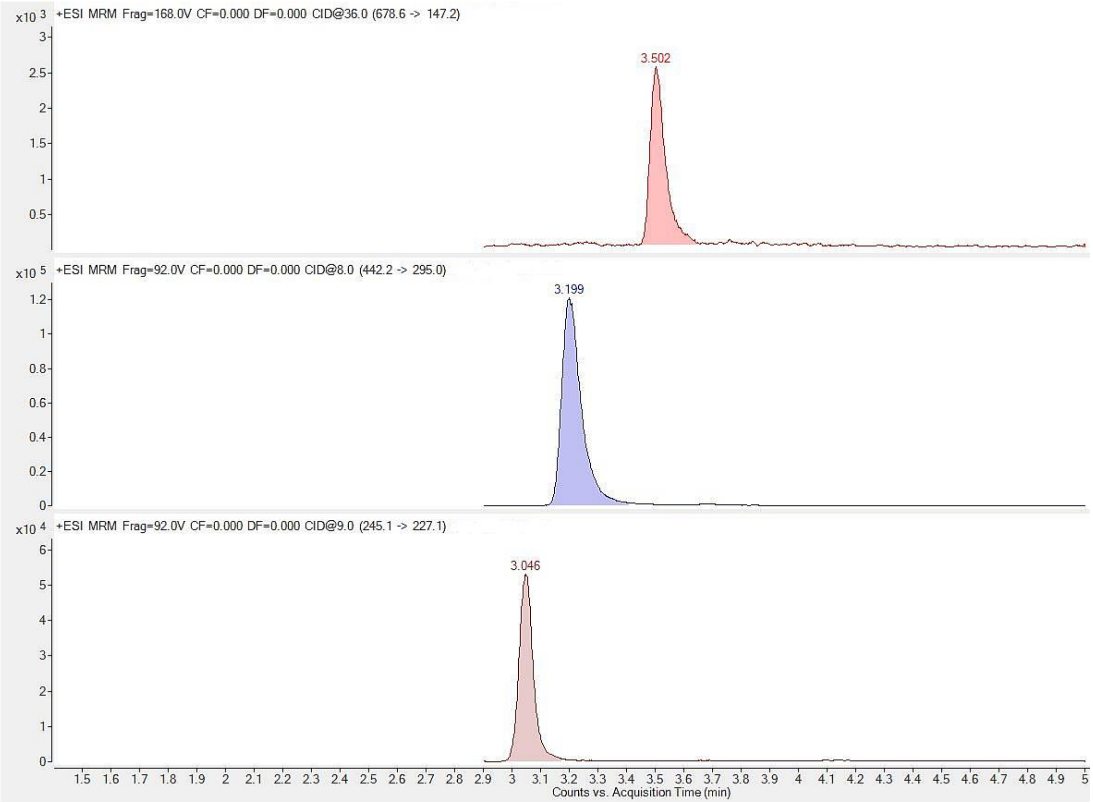

Figure S2. LC-MS/MS chromatogram obtained by the analysis of water-soluble vitamins in vitamin-fortified water. The top trace represents vitamin B12 (retention time 3.5 min); the middle trace represents vitamin B9 (retention time 3.2 min) and the bottom trace represents vitamin B7 (retention time 3.1 min).

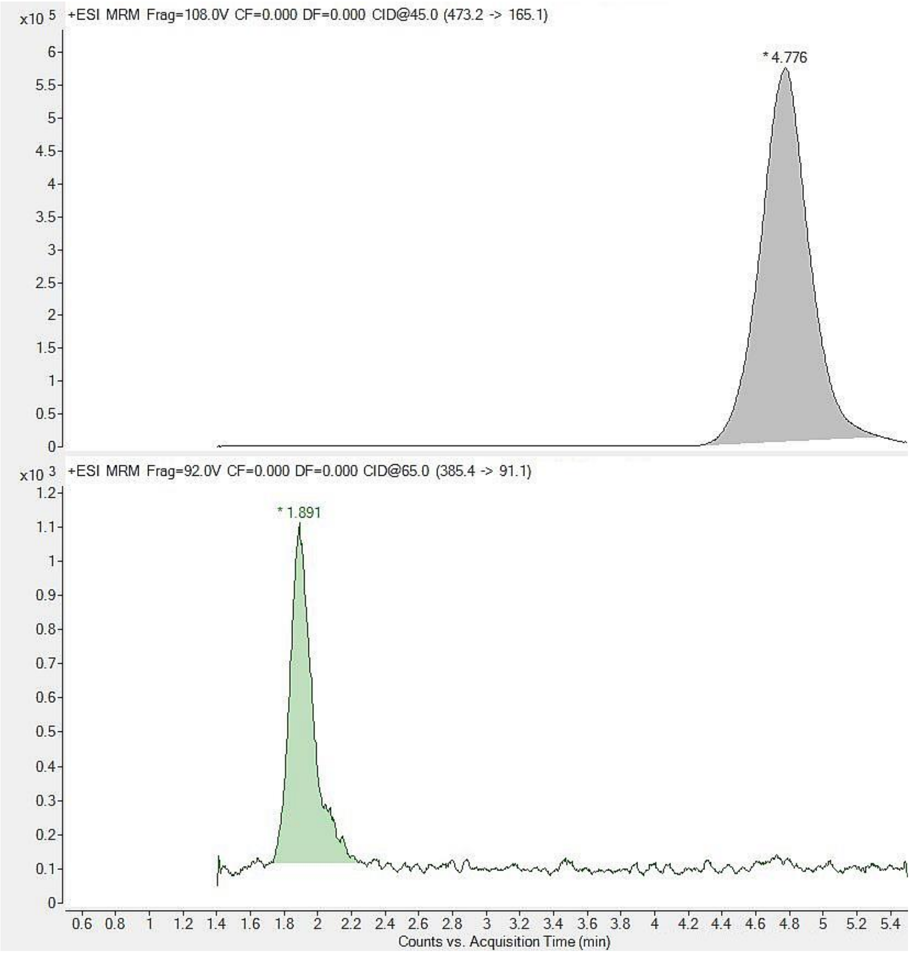

Figure S3. LC-MS/MS chromatogram obtained by the analysis of fat-soluble vitamins in vitamin-fortified water. The top trace represents vitamin E, in the form of alpha-tocopherol acetate (retention time 4.8 min), and the bottom trace represents vitamin D (retention time 1.9 min).

S5.2 Representative HPLC and LC-MS/MS chromatograms from the analysis of vitamin-fortified apple juice with numerical designation 33 in Figures 1, 3, and 6

a)

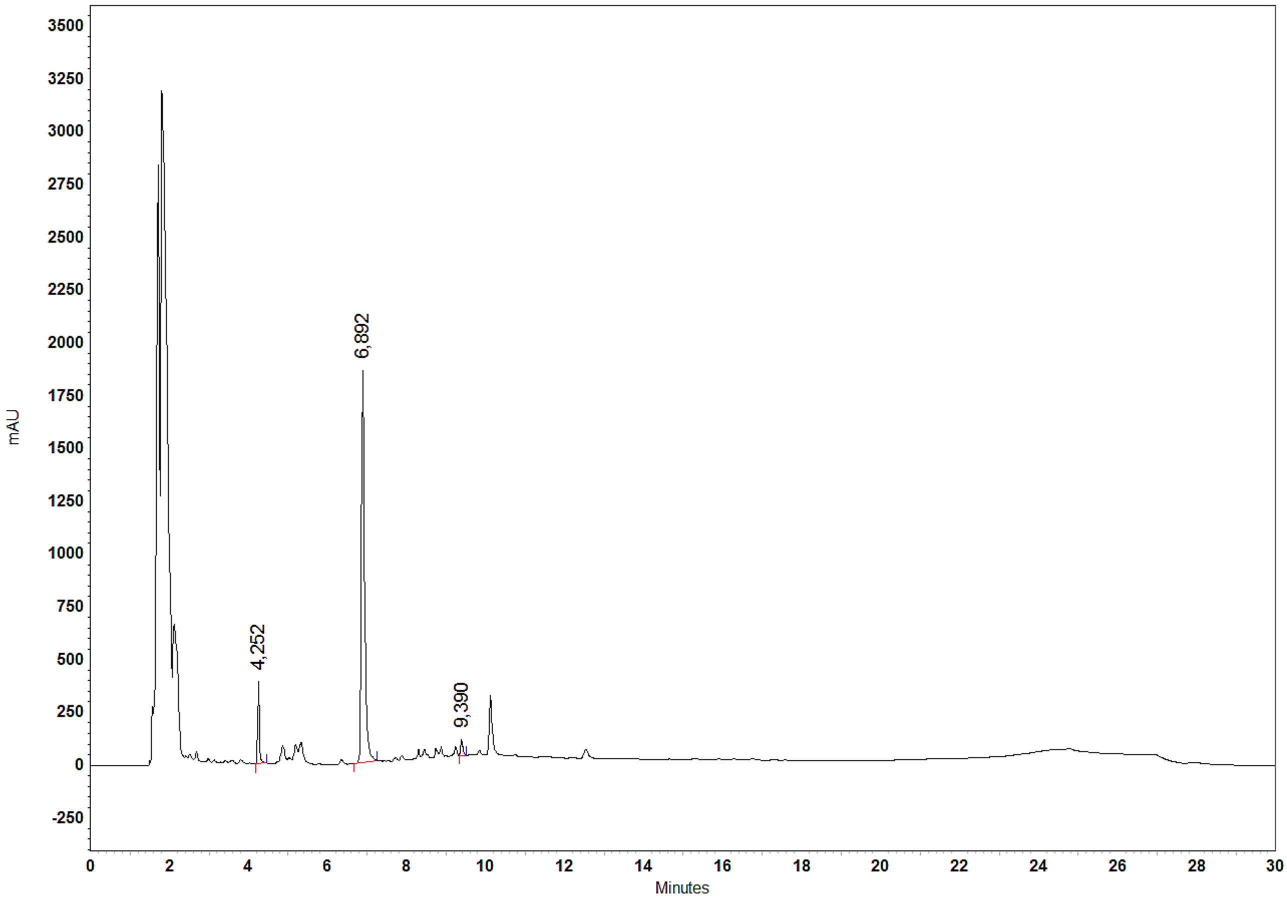

b)

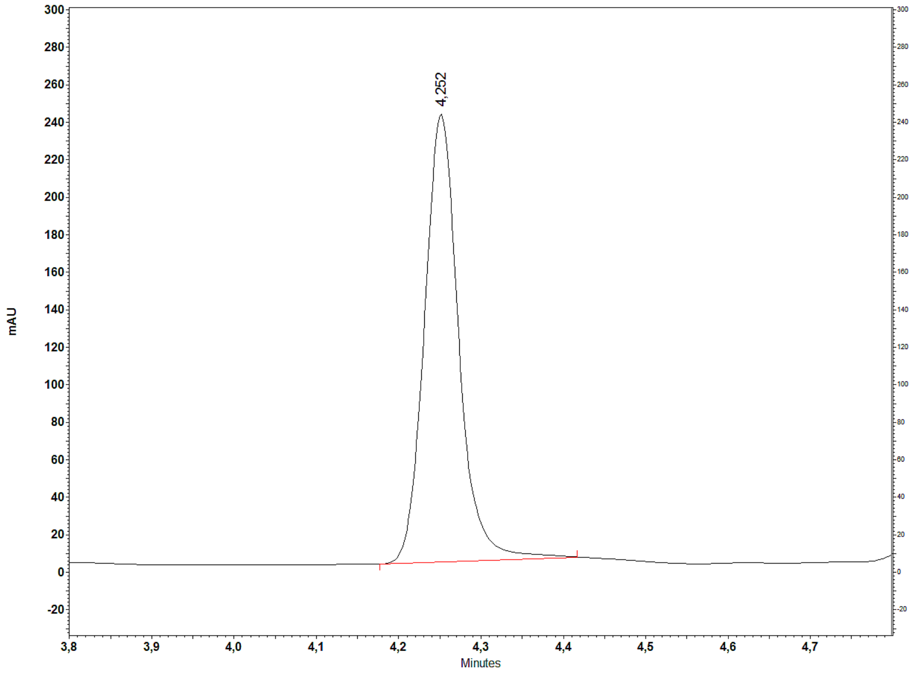

c)

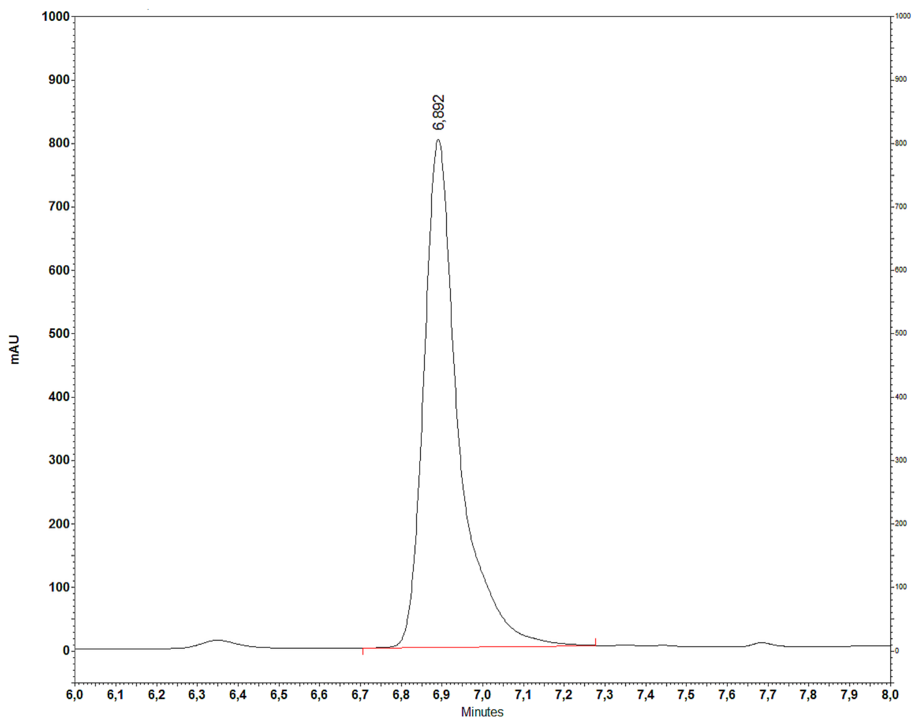

Figure S4. Representative HPLC chromatogram obtained by the analysis of water-soluble vitamins in vitamin-fortified apple juice at:
a) 210 nm - the wavelength used for the detection of vitamin B5 also representing the chromatographic separation of vitamins B6 (retention time 4.3 min), B3 (retention time 6.9 min), and B5 (retention time 9.4 min);
b) 290 nm - the wavelength used for the detection of vitamin B6;
c) 260 nm - the wavelength used for the detection of vitamin B3.

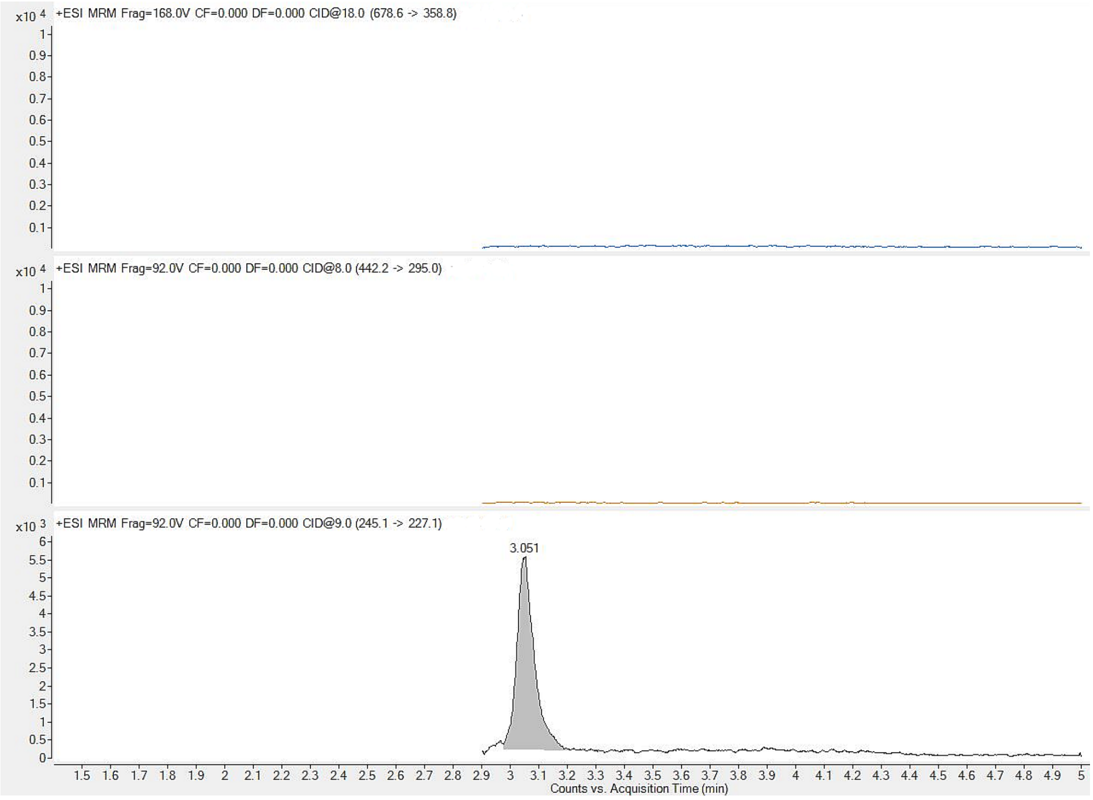

Figure S5. LC-MS/MS chromatogram obtained by the analysis of water-soluble vitamins in vitamin-fortified apple juice. The top trace represents the absence of the labeled vitamin B12 (retention time 3.5 min); the middle trace represents the absence of vitamin B9 (retention time 3.2 min) and the bottom trace represents vitamin B7 (retention time 3.1 min).

S5.3 Representative HPLC and LC-MS/MS chromatograms from the analysis of vitamin-fortified energy drink with numerical designation 44 in Figures 1, 3 and 6

a)

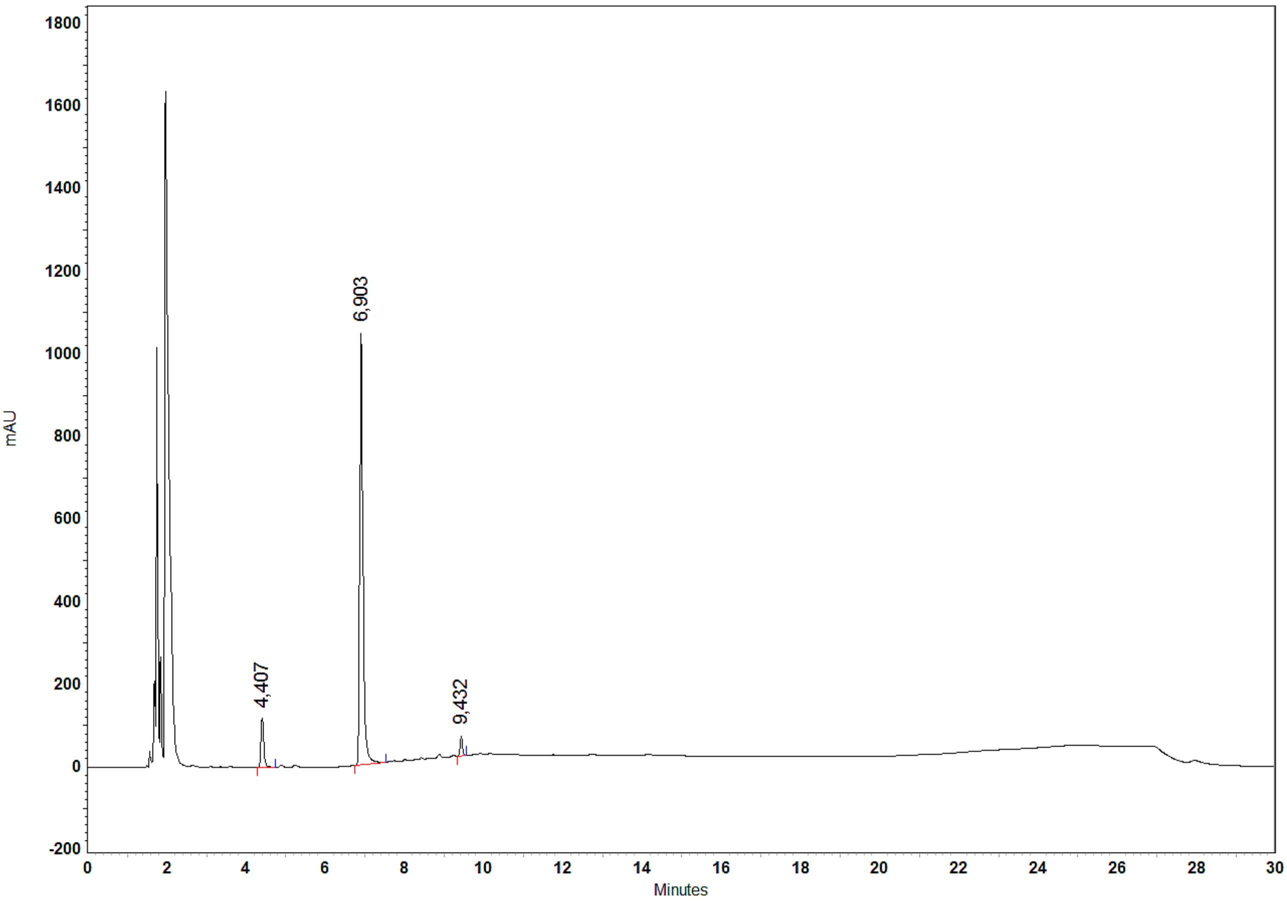

b)

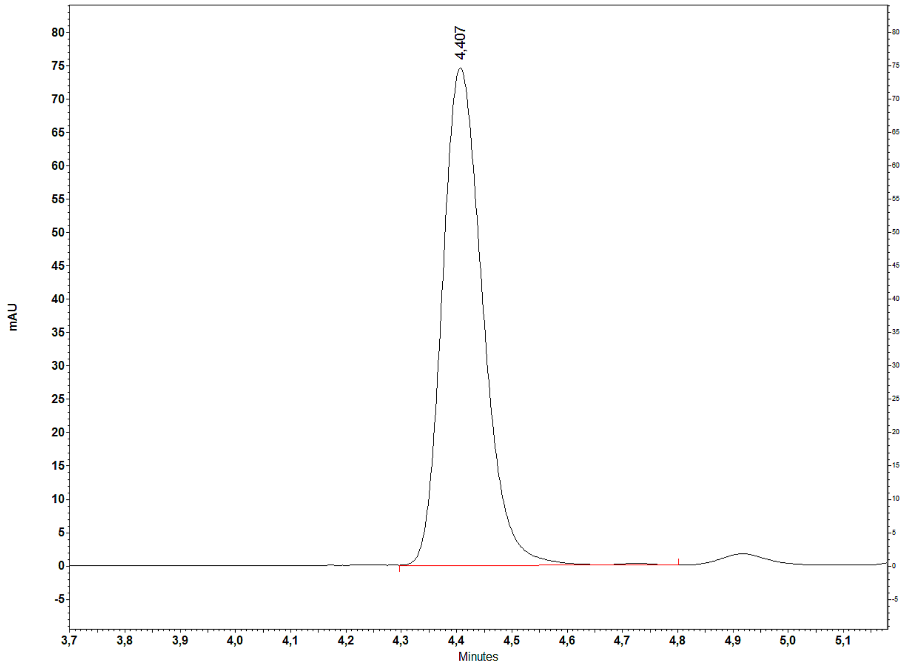

c)

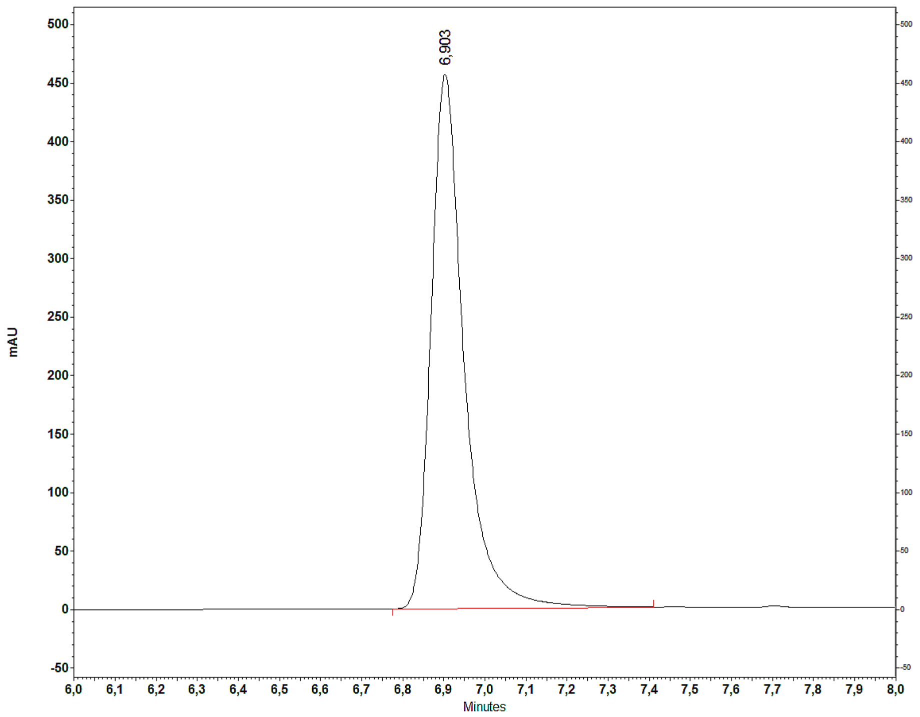

Figure S6. Representative HPLC chromatogram obtained by the analysis of water-soluble vitamins in vitamin-fortified energy drink at:

a) 210 nm - the wavelength used for the detection of vitamin B5 also representing the chromatographic separation of vitamins B6 (retention time 4.4 min), B3 (retention time 6.9 min), and B5 (retention time 9.4 min);

b) 290 nm - the wavelength used for the detection of vitamin B6;

c) 260 nm - the wavelength used for the detection of vitamin B3.

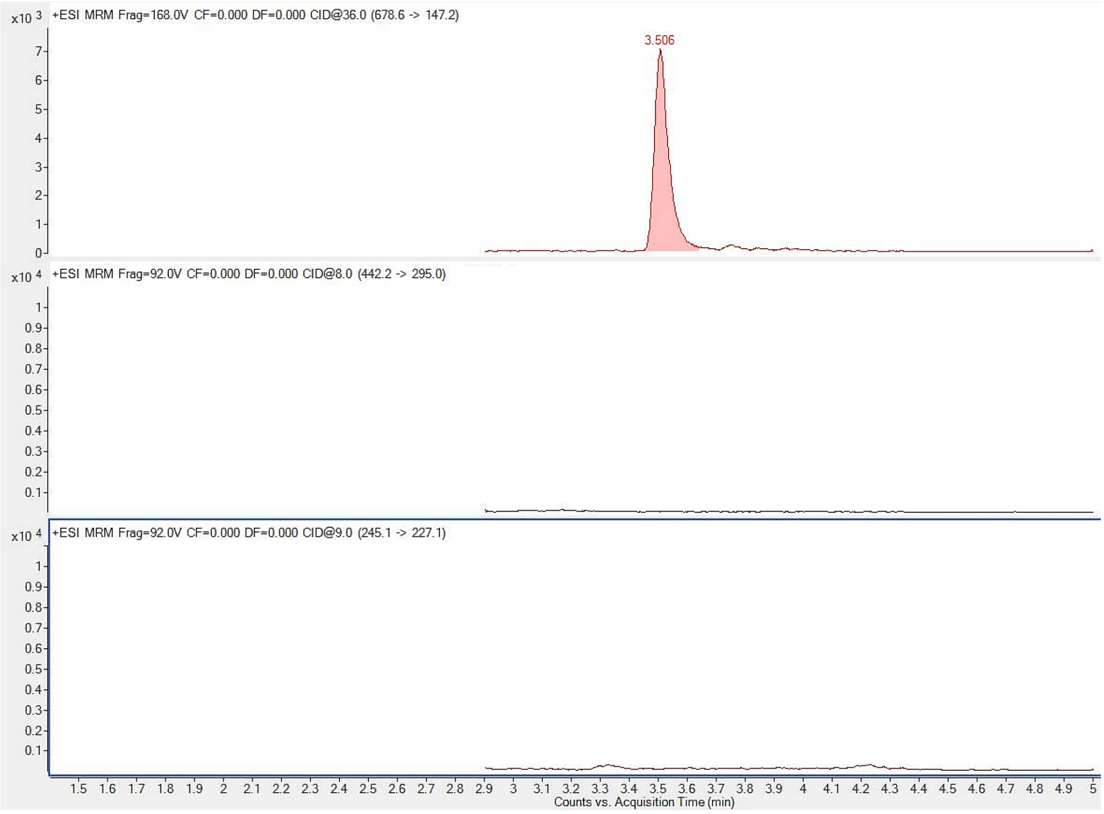

Figure S7. LC-MS/MS chromatogram obtained by the analysis of water-soluble vitamins in vitamin-fortified energy drink. The top trace represents vitamin B12 (retention time 3.5 min); the middle trace represents the absence of vitamin B9 (retention time 3.2 min) and the bottom trace represents the absence of vitamin B7 (retention time 3.1 min).
